# Effects of analgesic and noise stimulus in gait score assessment

**DOI:** 10.1371/journal.pone.0208827

**Published:** 2019-01-03

**Authors:** Ibiara Correia de Lima Almeida Paz, Ianê Correia de Lima Almeida, Elisane Lenita Milbradt, Fabiana Ribeiro Caldara, Marcos Livio Panhoza Tse

**Affiliations:** 1 Department of Animal Production, School of Veterinary Medicine and Animal Science (FMVZ), São Paulo State University (UNESP), Botucatu, São Paulo, Brazil; 2 School of Agrarian Science, Federal University of Grande Dourados, Dourados, Mato Grosso do Sul, Brazil; Tokat Gaziosmanpasa University, TURKEY

## Abstract

This experiment was carried out aiming to assess walking manner and speed of broiler chickens with different gait scores (GS), with or without sound stimulus, and with or without administration of analgesic. To that end, 1,000 birds were evaluated by the GS test and 74 were selected for walking speed analyses. Weight at slaughter and breast yield values were obtained for comparisons. Walking speed analyses, both with and without analgesic and with and without stimulus were performed. Non-parametric statistics was applied to the GS data that did not meet the assumptions of the statistical model (normality and homogenicity) using Fisher’s exact test according to the data behavior (P<0.05). The analyses of data on speed, weight at slaughter, and breast yield were evaluated by ANOVA and compared by Tukey’s test (P<0.05). Walking speed differed after acoustic stimulus with or without administration of metamizole sodium. Body weight was also different in each GS. It is thus concluded that the birds may feel discomfort when their GS is higher than 0, but that such discomfort may be suppressed when they are stimulated to walk.

## Introduction

High-yield broiler chickens have high incidence of leg issues [1]. Researchers have shown the animals have little ability to respond to the mechanical loads entailed by their conformation and weight when walking, which suggests the animals are not able to adapt their skeletal system as quickly as body weight increases [2, 3].

Improper skeletal system formation, which also results in locomotion issues, may decrease the well-being of broiler chickens due to reduced mobility. Chicken well-being is one of the main requirements of consumers, as are environmental conservation, quality standards, and food safety [4]. Therefore, animal well-being has become part of the marketing strategy of the food industry and there is growing need to use methods to estimate chicken comfort.

Locomotion disorders are commonly found issues in the production cycles of broiler chickens and cause high production losses. Walking difficulty is directly related to the well-being of broiler chickens since restrictions to freely walk around cause difficulty in satisfactorily eating, accessing drinking troughs, escaping threats, and expressing their natural behavior [5].

Based on the need for a methodology to measure animal well-being in the poultry industry, the gait score was created for help to estimate well-being through lameness. Some factors may impact the way chickens walk, among which fear of something new in the environment, aviary bedding material, hunger, and thirst [3,6,7,8,9]. Its advantage lies in that fact it can be employed in the stable [6–7] besides representing a non-invasive evaluation of a large number of birds within a short timeframe [10]. The current literature provides evidence that the leg birds suffer pain when walking [4–11,12]. Thus, application of analgesics improved the walk ability of the thigh birds [12], and to be a good indication of improvement in the welfare of these birds. In recent decades, gait score indices have worsened due to breeding for high breast deposition in modern broiler chickens, which resulted in changes in the center of gravity of birds with altered posture and angulation and, consequently, in prostrate position, which is likely the most comfortable position and that requires the least energy expenditure for birds with locomotive pathologies [8–11].

One of the techniques commonly used in commercial flocks to evaluate the gait score is the sound stimulus, which can negatively affect the test. However, this methodology is not described in the literature. The present study was carried out aiming to investigate the influence of gait scores on the walking speed of broiler chickens at the end of the fattening, with or without sound stimulus, and with or without administration of analgesic, because birds with leg problems suffer pain when they walk, however they overcoming this pain situation when with fear or under the effect of drug.

## Materials and methods

The trial was carried out at the area of teaching, research and extension in Broiler Farming of the School of Veterinary Medicine and Animal Sciences (FMVZ) of the São Paulo State University–campus Botucatu, after being submitted to, evaluated, and approved by the Animal Use Ethics Committee of FMVZ under protocol no. 113/2017.

### Birds and experimental management

The study was carried out a masonry experimental poultry barn with concrete floors covered with wood shavings and no boxes, density 12 birds/m^2^. The facility featured automated nipple drinking troughs, automated helicoidal feeding troughs, and inner and outer curtains. In negative pressure, temperature was controlled by exhaust fans and evaporative panels, that were automatically activated as needed to maintain the temperature determined for each age in the linage manual. Between the first and seventh days of age, an automated gas turbine heater was used. For this experiment, 33% of the barn was longitudinally delimited with one row of feeding troughs and two rows of drinking troughs. All birds were fed same feed and water *ad libitum*. Feeding was split into five production phases: pre-initial (one to seven days, 22% Crude Protein and 2850 kcal ME/kg), initial (eight to 14 days, 21,5% CP and 2950 kcal ME/kg), growth I (15 to 21 days, 19,5% CP and 3050 kcal ME/kg), growth II (22 to 35 days, 19% CP and 3100 kcal ME/kg), and final (36 to 43 days, 18,3% CP and 3180 kcal ME/kg).

### Gait score

At 41 days of age, the gait score was assessed by a trained observer according to the methodology proposed [9], when all birds were classified according to their locomotion ability.

A three-point scale was applied to classify the gait score of the birds. Score 0 (GS 0) was attributed to healthy birds that exhibited no abnormality when walking; score 1 (GS 1), to birds that exhibited difficulty to walk that impacted their locomotion ability in a way that was easily identifiable through observation; and score 2 (GS 2), to birds that exhibited severe issues and walked only when highly motivated.

At the beginning of the experiment 1000 birds were housed, there was 3% mortality, therefore at 41 days the gait score was evaluated in 970 birds. Based on the test results, 74 birds that precisely represented each score (46 with GS 0, 23 with GS 1, and five with GS 2) were chosen for assessment of walking speed. This was the maximum number of birds possible to be assessed at the same day, due to the large number of video image catches, so that the animals not be stressed and the study maintain the quality, then the total birds was found for the 2 and 3 gait scores and those possible for GS 0 were assessed. Still at the aviary, one day before the videos were taken, the body weight of each bird was measured using a balance with 1.0 g precision.

### Experimental Design

The study employed a completely randomized 3 x 2 x 2 factorial design for a total of 12 treatments, namely, three gait scores (0, 1, and 2), with or without stimulus, and with or without application of analgesic.

### Walking speed–image analysis

Video images were recorded after the gait score test was carried out on all birds in the study. The number of replicates was not determined since the birds were chosen under strict criteria aiming to clearly show the particular characteristics of each gait score when walking.

**Fig 1:**
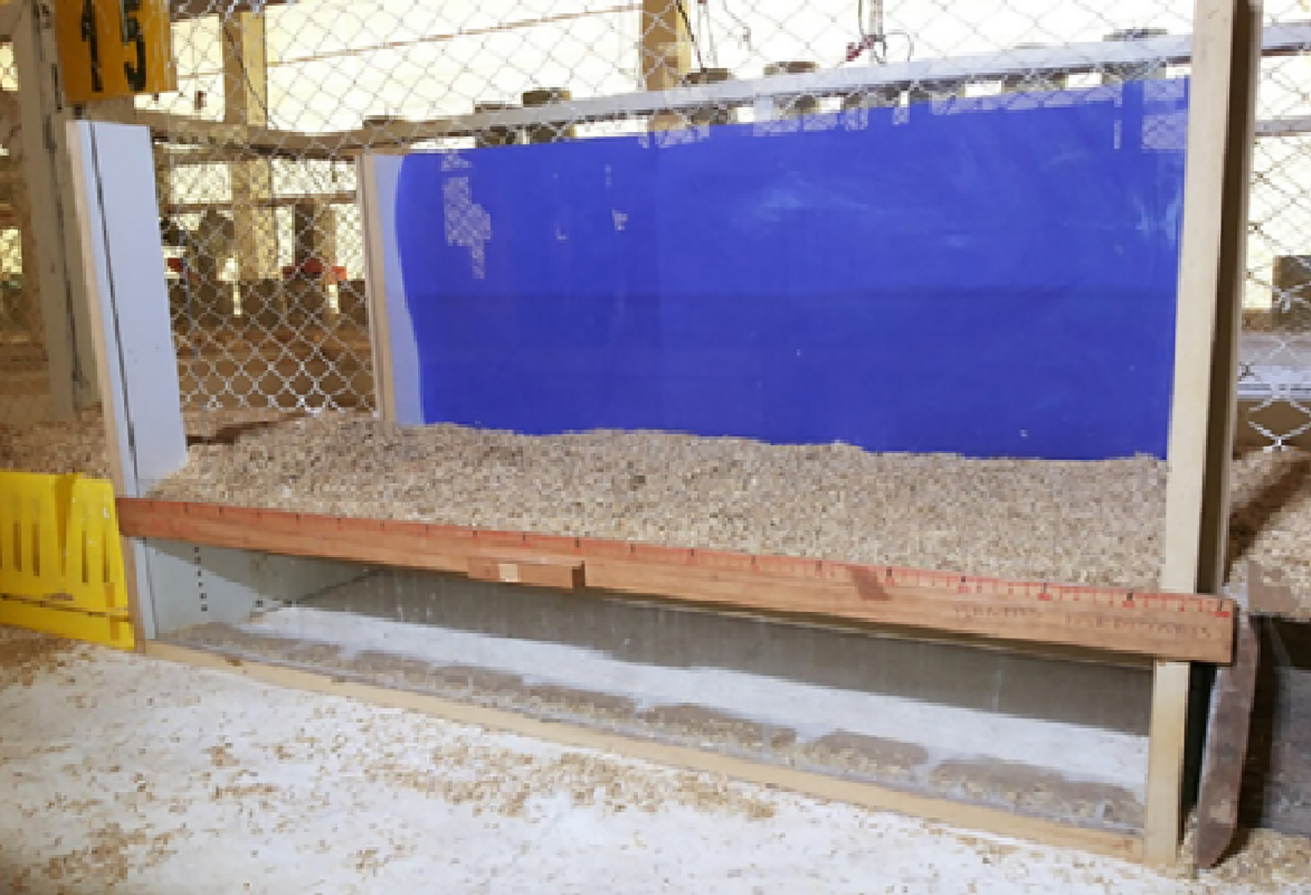
Test area 1 m x 30 cm, flat surface, and 8 cm deep aviary bedding (wood shavings), 50 cm tall acrylic plates were placed at the sides of the track.

The images were recorded at the test area, whose layout had 1 m x 30 cm, flat surface, and 8 cm deep aviary bedding (wood shavings) to reflect the conditions found at the rearing environment. In order to delimit the test area and allow images to be taken with little variation in distance, 50 cm tall acrylic plates were placed at the sides of the track (Fig 1).

The selected birds were submitted to a new gait score test, this time recorded in video. The camera used to capture the images was a Sony HDR-PJ200 Handycam equipped with Vario-Tessar Carl Zeiss lenses. The videos were recorded in AVI format with 1920 x 1080 resolution and rate of 60 frames per second. The camera was mounted on a tripod 1 m from the test area at 15 cm height.

The captured images allowed measuring the speed (cm/s) reached by the birds when walking as a function of the different treatments used.

The images were evaluated in two ways. First, the speed of the birds over the full track (1 m) was assessed in the different treatments. Then, their acceleration was verified by calculating the mean speed over the first 50 cm and in the remaining course while attributing a mean speed for each half of the track.

### Stimulation and analgesic administration

After that, the selected birds were stimulated to walk by noise and the images were captured again. The noise used as sound stimulus was the clap hands, reaching between 60 and 80 dB. During the third stage, the birds were administered an oral rapid-onset analgesic (metamizole sodium) according to the methodology employed by [12]. This medication is used in several countries as a human analgesic and is employed in animal studies. The amount of analgesic administered to the birds was calculated proportionally to the body weight (1 drop per kg live weight). That the drug was administered with high accuracy, it was necessary to carry out their dilution in distilled water, so, every drop was diluted in 1ml of water, and was possible to admise exact doses of the analgesic as a function of the body weight of broilers. According to Prati Donaduzzi [13], the analgesic and antipyretic effects of metamizole sodium can be expected within 30 to 60 min after administration and usually last for about 4 h. After 60 min, the video gait score test was carried out again with and without stimulus to walk.

### Body weight and breast yield

At the age of 42 days, the birds were transported to the experimental slaughterhouse of FMVZ–UNESP, individually weighed on a balance with 1.0 g precision, stunned by electronarcosis, and then slaughtered by bloodletting through a cut at the jugular vein and carotid arteries. The whole breast (with bone, muscle, and skin) was weighed and its percentage in relation to body weight was calculated.

### Statistical analysis

The results were analyzed using the statistical software SAS 9.2. The variance homogeneities were assessed by Levene’s test and data normality was verified by Shapiro-Wilk test.

The gait score data were shown by the frequency procedures and Fisher’s test (P<0.05) was applied. The body weight, breast weight, breast yield, and walking speed were evaluated by ANOVA and their means were compared by Tukey’s test (P<0.05).

## Results and discussion

The mortality found for birds in this study was 3%, thus the gait score frequency results are based on 970 broiler chickens (Table 1). The other characteristics presented in this study refer to the number of birds selected for image evaluation, i.e., GS 0 = 46 birds, GS 1 = 23 birds, and GS 2 = 5 birds.

**Table 1:** Gait score frequency, body weight, and breast weight and yield of broiler chickens at 41 days of age.

Gait score	Gait score frequency (%)	Body weight (g)	Breast weight (g)	Breast yield (%)
0	89.07 A	2750 B	841 A	30.60
1	9.90 B	2931 A	926 A	31.61
2	1.03 C	1897 C	607 B	31.96
Mean	-	2526	791	31.39
CV%	-	14.87	13.56	2.31
P value	0.015	0.036	0.048	0.985

CV: coefficient of variation. Means followed by different capital letters in the column differ according to Fisher’s test for gait score frequency and by Tukey’s test for body weight, breast weight, and breast yield, both at 5% probability.

The GS frequencies were similar to those found in studies by other authors [5,8,14,15]. Checking the relevant literature was found studies with 3 point gait score and gait score of six points, it is known that this variation is closer to the accuracy wished [12], in the industry evaluations is not so utilize six points. However, it is possible to compare the research regardless of the scale used, for it is known that the minor scores refer to the birds that walk better and the higher scores refer to those birds that walk worse. Likewise, breast yield did not differ (P>0.05) among different GS groups, however, body weight and breast weight did differ (P<0.05) among the birds (Table 1). The literature also reports such differences in absolute weights and not yield, i.e., in percentage values [14,16].

Another noteworthy detail is that animals in GS 1 had the highest body weight, which shows that first weight gain may impact the way the birds walk, however, as GS worsens by the end of the production cycle, no deleterious effect to the animal occurs. The lowest body weight was found for GS 2 chickens, which leads to the conclusion that those birds had locomotion issues since the earliest rearing phases, thus hindering their access to feeding troughs and drinking troughs and impairing their performance.

**Fig 2:**
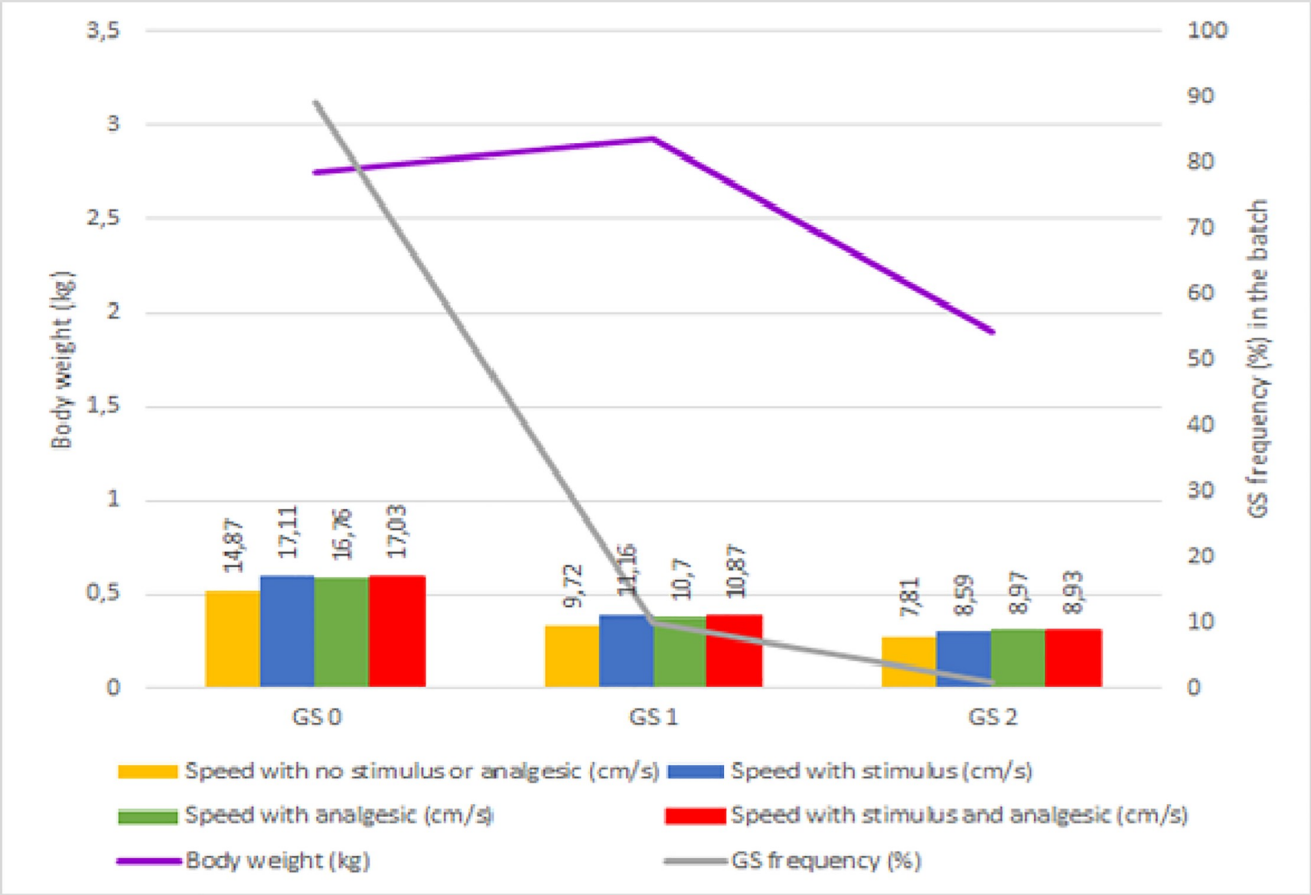
Frequency of gait score, speed of broilers chickens at 41 days of age, body weight at 40 days of age of broilers with or without stimulus, and with or without application of analgesic. Score 0 (GS 0): Healthy birds that exhibited no abnormality when walking; Score 1 (GS 1): Birds that exhibited difficulty to walk that impacted their locomotion ability in a way that was easily identifiable through observation; Score 2 (GS 2): Birds that exhibited severe issues and walked only when highly motivated.

No interaction (P>0.05) was found among the treatments. When bird speed in the different situations is assessed, it is seems that GS impacts (P<0.05) the speed of the chickens, with healthy birds walking faster than those with GS above 0 (Table 2 and Fig 2). The use of an acoustic stimulus, whether or not associated with metamizole sodium, increased the speed of broiler chickens, which shows the birds can withstand discomfort to escape an aversive situation [17].

**Table 2:** Walking speed of broiler chickens at 41 days of age.

Gait score	Number of birds in each GS	Speed with no stimulus or analgesic	Speed stimulus	Speed analgesic	Speed with stimulus and analgesic	Mean	CV (%)	P value
		(cm/s)						
0	43	14.87	17.11	16.76	17.03	16.44 A	8.34	0.037
1	26	9.72	11.16	10.70	10.87	10.61 B	7.63	0.042
2	5	7.81	8.59	8.97	8.93	8.57 C	7.25	0.035
Mean		10.80 b	12.29 a	12.14 a	12.28 a			
CV%		9.63	10.53	9.25	9.00			
P value		0.038						

CV: coefficient of variation. Means followed by different capital letters in the columns and different small letters on the row differ according to Tukey’s test at 5% probability.

Therefore, it is important to verify that, when the gait score test is applied at commercial farms, the parameter “fear” must be taken into account since the results may be masked if the birds are stimulated.

Walking speed is a commonly assessed parameter in studies on locomotion difficulties [18,19]. This is a successful strategy to show the influence of pain on gait and mostly improvements after analgesic administration can be observed [20].

Similar results were reported by [12], who assessed the gait score of broiler chickens under natural conditions and after analgesic administration. That study found a reduction in the time the birds took to complete the test, particularly when they had intermediate and poor scores, after drug administration. Those same authors concluded that providing analgesics to lame birds improved their performance and speed in the gait score test even to the point of reducing their scores, which shows that birds with locomotion disorders experience pain and, consequently, have reduced well-being. In their study, [21] assessed the administration of analgesic drugs mixed with the feed and found that the walking speed of broiler chickens with locomotion disorders improved after they consumed the feed, which indicated pain relief. To [20] broiler chickens free of locomotion disorders raise their legs more when walking than lame ones. That was shown by providing analgesic drugs to the birds, whose walking improved after the treatment.

It should be emphasized that the birds were submitted to the gait score test in several moments (with and without sound stimulus, with and without analgesic), so this evaluations lasted all day, for the 74 birds, being carried out quite calmly.

**Fig 3:**
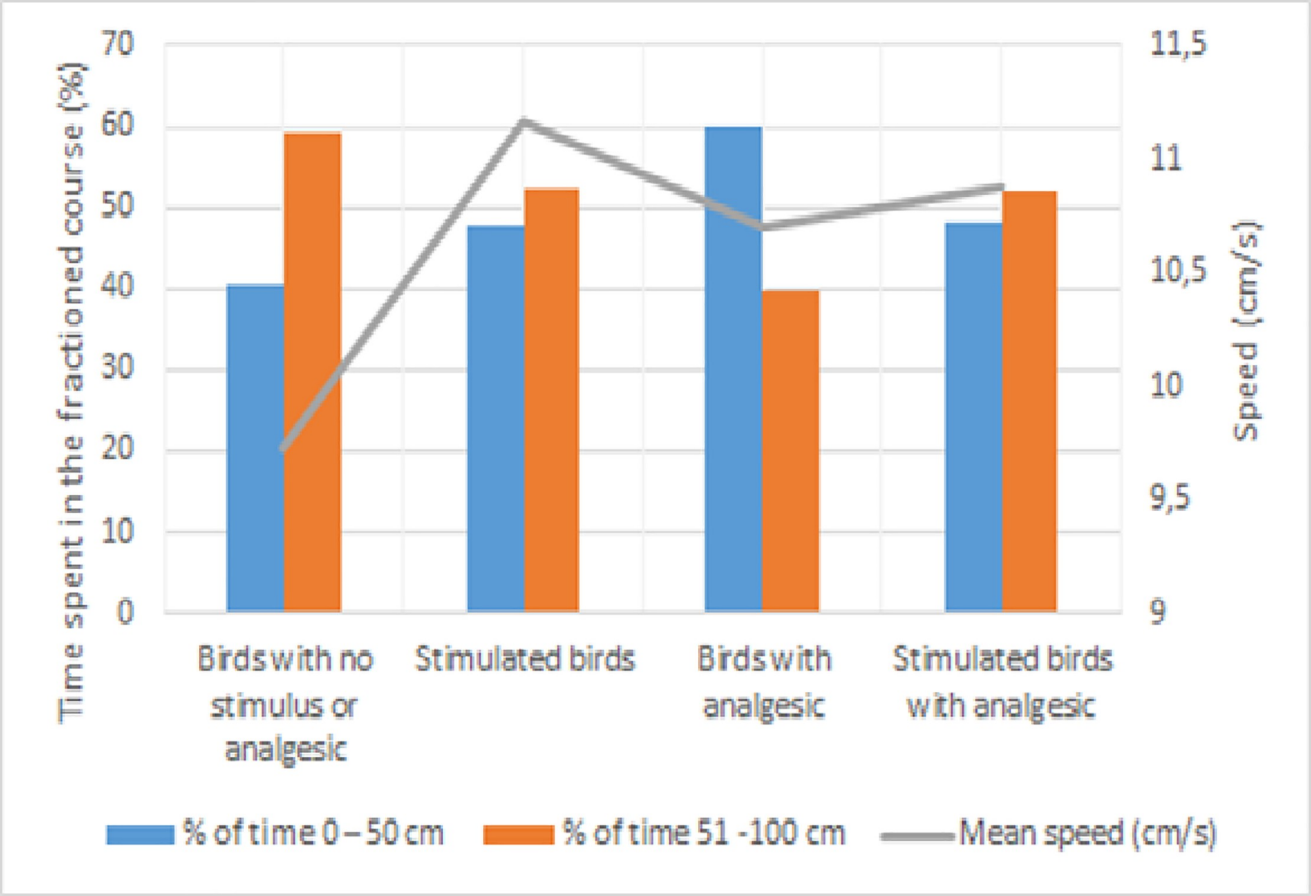
Speed (cm/s) and time distribution (%) among each animal group to finish the course of 1 m linear course.

By fractioning the 1 m linear course into two parts, it could be seen that the mean speeds of the birds are different in the first and second halves (Table 3 and Fig 3), which resulted in different distributions of time among each animal group to finish the course. The birds that received no stimulus or analgesic medication had better speed in the first 50 cm than in the second half of the track, whereas stimulated birds (with or without analgesic administration) had virtually constant speed and the chickens that received analgesic had better speed in the final 50 cm. That may be related to pain memory, which makes the animal take a few seconds to realize the pain is not present anymore. Pain is defined as an unpleasant sensory and emotional experience, however, it is essential given its defensive character [22]. Similar reports have been made on humans with chronic pain, who take longer than others with no such condition to respond to stimuli [23]. Fear is a protective mechanism that causes the animal to respond to potentially harmful stimuli. The behavioral responses can include escape and avoidance behavior, defensive behavior, freezing, immobility and vocalizations [24].

**Table 3:** Distribution of speed (cm/s) and percentage of time for each category of broiler chickens, the first and second half of the course.

Course	Birds with no stimulus or analgesic	Stimulated birds	Birds with analgesic	Stimulated birds with analgesic
	Speed (cm/s) and percentage of time spent in the course			
0–50 cm	23.91 cm/s (40.65%)	23.40 cm/s (47.69%)	17.79 cm/s (60.13%)	22.53cm/s (48.23%)
51–100 cm	16.37 cm/s (59.35%)	21.33 cm/s (52.31%)	26.84 cm/s (39.87%)	21.00 cm/s (51.77%)

When in fear, the birds apply more force than usual to walk, perhaps due to stress and their flight mechanisms. In humans, this response can be called reaction speed, using maximum explosive force, which is the capacity of obtaining elevated force values within a very short time. Such force is reached when the individual is submitted to stress or undergoes rigorous training, however, it lasts for a few seconds [25].

As previously described, the medication used in this study was metamizole sodium, which has about 66% greater antipyretic and analgesic action compared to other medications such as paracetamol and acetylsalicylic acid [26]. This medication acts on the central nervous system by desensitizing peripheral nociceptors and inhibiting the perception of pain by the patient [27,28]

## Conclusion

The walking speed of birds in each gait score was different and improved after analgesic administration, mainly for birds with GS higher 0, which allows concluding that their discomfort decreases with medication. However, acoustic stimulation of the birds, whether or not associated with analgesic use, also led to improved acceleration of the birds in each course. That leads to the conclusion that the fear caused by this stimulus overcomes the discomfort caused by locomotion issues.
